# The Legacy of COVID-19 in Breast Milk: The Association of Elevated Anti-Inflammatory and Antimicrobial Proteins with Vaccination or Infection

**DOI:** 10.3390/cimb47030182

**Published:** 2025-03-11

**Authors:** Felicia Trofin, Petru Cianga, Daniela Constantinescu, Luminița Smaranda Iancu, Roxana Irina Iancu, Diana Păduraru, Eduard Vasile Nastase, Elena Roxana Buzilă, Cătălina Luncă, Corina Maria Cianga, Olivia Simona Dorneanu

**Affiliations:** 1 Microbiology Discipline, Preventive Medicine and Interdisciplinarity Department, University of Medicine and Pharmacy “Grigore T. Popa”, 700115 Iasi, Romania; felicia.trofin@umfiasi.ro (F.T.); luminita.iancu@umfiasi.ro (L.S.I.); elena-roxana.buzila@umfiasi.ro (E.R.B.); catalina.lunca@umfiasi.ro (C.L.); olivia.dorneanu@umfiasi.ro (O.S.D.); 2 “Sf. Spiridon” Clinical Hospital, 700111 Iasi, Romania; daniela.constantinescu@umfiasi.ro (D.C.); roxana.iancu@umfiasi.ro (R.I.I.); corina.cianga@umfiasi.ro (C.M.C.); 3 Immunology Discipline, Ist Morpho-Functional Sciences Department, University of Medicine and Pharmacy “Grigore T. Popa”, 700115 Iasi, Romania; 4 Iasi Regional Center for Public Health, National Institute of Public Health, 700465 Iasi, Romania; 5 Phisiopathology Discipline, IInd Morpho-Functional Sciences Department, University of Medicine and Pharmacy “Grigore T. Popa”, 700115 Iasi, Romania; 6 “Dr. C.I. Parhon” Clinical Hospital, 700503 Iasi, Romania; diana_paduraru@email.umfiasi.ro; 7 Clinical Hospital of Infectious Diseases “Sf. Parascheva”, 700116 Iasi, Romania; eduard-vasile.nastase@umfiasi.ro; 8 Infectious Diseases Discipline, Medical Sciences II Department, University of Medicine and Pharmacy “Grigore T. Popa”, 700115 Iasi, Romania; 9 “Sf. Maria” Children Emergency Hospital, 700309 Iasi, Romania

**Keywords:** COVID-19, SARS-CoV-2, breastfeeding, lactoferrin, lactadherin, furin, tenascin C, granzyme B, chitinase 3-like 1, antimicrobial proteins

## Abstract

Background: Breast milk is a rich source of antimicrobial and anti-inflammatory compounds, owing to its diverse array of bioactive molecules. This study explores the presence and activity of natural antimicrobial agents in breast milk, particularly in the context of the SARS-CoV-2 pandemic. Materials and Methods: Breast milk samples were collected from 50 breastfeeding mothers, including those who had either been vaccinated against SARS-CoV-2 or had recovered from the infection. These samples were compared with a control group consisting of 10 unvaccinated mothers with no history of COVID-19. Key antimicrobial and immune-regulatory proteins—lactoferrin, lactadherin, furin, tenascin C, granzyme B, and chitinase 3-like 1—were quantified using the Luminex multiplex analyzer. Results and Discussion: All targeted biomarkers were detected in breast milk, providing insights into the immune profile transferred to infants following COVID-19 infection or vaccination. These bioactive molecules highlight breastfeeding’s role in providing passive immunity and antimicrobial protection. The protein levels were found to be influenced by factors such as maternal inflammation, infant age, delivery mode, and parity, emphasizing the dynamic interaction between maternal immunity, lactation biology, and infant development. Conclusion: Breastfeeding serves as a powerful anti-SARS-CoV-2 defense mechanism, supported by the activity of lactoferrin, lactadherin, and furin, reinforcing its critical role in child health.

## 1. Introduction

Breastfeeding is a cornerstone in supporting the survival, nutritional needs, and developmental progress of infants and young children while also promoting maternal health [1,2].

The properties of breast milk that protect the infant from a wide range of diseases, particularly infections, are well documented (Table 1).

The effectiveness of breast milk is attributed to its diverse array of bioactive molecules that have been shown to offer protection against infections [20,21], reduce inflammation [22], enhance immune function [23], and influence the infant microbiome [14,24]. These benefits are particularly vital during infancy when the innate immune system is still developing. In response to the emergence of novel viruses that can cause pandemics and the increased vulnerability of specific populations to severe infections, both national and international research initiatives are focused on identifying new antimicrobial agents or natural substances with potential efficacy against infections. In this context, breast milk has gained significant attention, becoming the subject of numerous studies. Preclinical evaluations of human milk are increasingly being translated into clinical applications, with the possibility of a large-scale production of its active compounds [25].

In lactating mothers who have been immunized, IgG and IgA antibodies have been identified in breast milk [26], offering passive immunity to the infant and safeguarding against infections during the first year of life [27]. In the event of an infection in either the mother or the infant [28], breast milk serves as a source of numerous antipathogenic and anti-inflammatory bioactive factors that contribute to the infant’s immune defense [29].

The mother’s initial response to infection contributes specific immunological factors to breast milk, which could help prevent infection or mitigate the severity of the illness. In the breast milk of mothers infected with various microorganisms, a range of immune and anti-inflammatory biomarkers, including immunoglobulins A (IgA) and G (IgG) [8], cytokines and chemokines [30], as well as enriched levels of lactadherin, butyrophilin, and xanthine dehydrogenase, have been identified [31].

The cultivation of viruses from RNA-positive breast milk samples collected from infected mothers appears to be unachievable as no replication-competent virus has been detected in any of the samples, including those that tested positive for viral RNA. These findings indicate that the virus particles identified in breast milk may not be capable of causing infection [32,33,34,35]. Even if the infectious agent is excreted in breast milk, evidence suggests that it does not lead to infections in infants [36,37,38,39].

Recent studies conducted during the COVID-19 pandemic suggest that breastfeeding may exert an inhibitory effect on SARS-CoV-2. Furthermore, research indicates that SARS-CoV-2, SARS-CoV, and Middle East respiratory syndrome are not transmitted via breast milk [40,41,42,43].

Aim of the Study: Numerous inquiries have emerged regarding the potential transmission of the SARS-CoV-2 virus or vaccine components to infants or young children through breastfeeding by mothers who are infected with SARS-CoV-2 or vaccinated against COVID-19. While many studies have addressed and alleviated these concerns [8,30,44,45], the present research seeks to emphasize the beneficial effects of breastfeeding for infants of mothers affected by the virus or vaccinated against COVID-19. Consequently, our study aims to investigate whether bioactive components with antimicrobial properties, previously identified in various breast milk contexts, are secreted or show alterations in concentration in response to SARS-CoV-2 infection or immunization status.

In selecting the proteins for analysis, we focused on those known for their antimicrobial or anti-inflammatory roles, which have been studied in the context of COVID-19 and detected in breast milk under various conditions. Accordingly, we have chosen to assess six antimicrobial proteins—chitinase 3-like 1 (C3-1), furin (F), granzyme B (GB), lactoferrin (LF), lactadherin (LD), and tenascin C (TC)—in the breast milk of mothers infected with SARS-CoV-2 compared to those vaccinated against COVID-19.

To the best of our knowledge, there is limited data regarding the specific role of human milk in combating COVID-19. However, numerous studies have extensively documented its antimicrobial properties against various viral agents.

## 2. Materials and Methods

### 2.1. Research Methodology and Subjects

**Figure 1:**
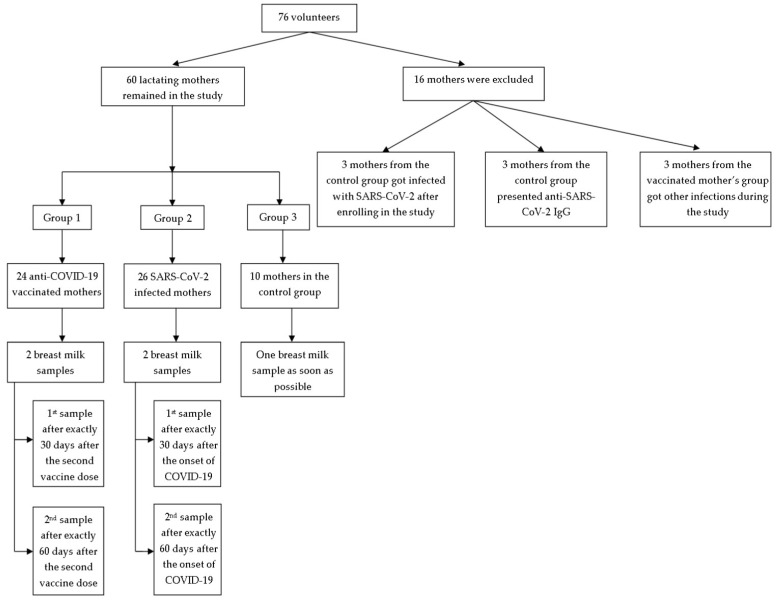
Study group flowchart.

This study outlines a prospective randomized cohort study conducted between February—May 2021 at the “Grigore T. Popa” University of Medicine and Pharmacy, Iasi, Romania, which aimed to determine the concentrations of lactoferrin, lactadherin, furin, tenascin C, granzyme B, and chitinase 3-like 1 in human breast milk. Three groups were formed based on the inclusion and exclusion criteria presented in Table 2: vaccinated mothers, infected mothers, and a control group (Figure 1).

Information gathered from participants included maternal age, date of childbirth, parity, mode of delivery, vaccination regimen, post-vaccination side effects, COVID-19 symptoms, and any subsequent hospitalizations. All participants voluntarily enrolled in the study after providing informed consent, which was specifically designed for this investigation. Mothers were trained on the proper technique for milk collection to avoid contamination, including using sterile containers, collecting from the middle stream, and immediately freezing the samples, as previously described by Trofin et al. (2022) [8].

The volunteers were closely monitored for 70 days, beginning from the onset of symptoms or the date of vaccination, up to 10 days following the second breast milk sample collection. This monitoring period aimed to observe whether the second sample coincided with the onset of any additional infections or inflammation that could influence the results. Similarly, the control group mothers were followed for 10 days after providing their samples to detect any infections or inflammations that could potentially affect the concentrations of the tested parameters.

The number of study participants was calculated using a sample size calculator. According to the sample size calculator, a minimum of 59 surveys was required to achieve a 95% confidence level, ensuring that the true value falls within ±5% of the observed value, thereby satisfying the specified statistical requirements.

### 2.2. Sample Collection

Participants were provided with sampling kits that included detailed instructions for the collection and storage of breast milk. Sterile tubes were used to collect 1 mL of breast milk, obtained from the middle flow. The samples were then immediately frozen at −20 °C and stored until further analysis. To maintain sample integrity, the breast milk was transported to the laboratory within 30 min in refrigerated containers, ensuring that the samples did not thaw.

### 2.3. Sample Analysis

Upon thawing, the samples were allowed to gradually reach room temperature and were then vortexed for 20 s. To remove milk fat, the tubes were centrifuged at 5000× *g* for 25 min at 4 °C. After centrifugation, the skimmed milk was carefully transferred to new tubes for further processing.

All breast milk samples were evaluated for the presence of reactive anti-S1 RBD IgG antibodies using a sandwich enzyme-linked immunosorbent assay (ELISA), as previously outlined by Trofin et al. (2022) [8]. A 1:10 dilution of the samples was prepared in the provided sample diluent, and the assay was performed following the manufacturer’s instructions (TestLine Clinical Diagnostics, Brno, Czech Republic, EIA COVID-19 RBD IgG, with a test specificity of 99.15% and test sensitivity of 99.9%). Optical densities were measured at 450 nm using a TECAN Infinite 200 photometer (Tecan Austria GmbH, Grödig, Austria), and the results were analyzed using Magellan Pro V7.4 software.

The same breast milk samples were used to quantify chitinase 3-like 1, furin, granzyme B, lactoferrin, lactadherin, and tenascin C by a bead-based immunoassay utilizing the Luminex Multiplexing Assay. The process followed the manufacturer’s guidelines (Human Premixed Multi-Analyte Kit, R&D Systems, Minneapolis, MN, USA). Sample analysis was conducted using a Luminex 200 device (Luminex Corporation, Austin, TX, USA). The antimicrobial proteins quantified included chitinase 3-like 1, furin, granzyme B, lactoferrin, lactadherin, and tenascin C.

The decision to quantify the six parameters using the Luminex multiplexing assay was driven by its multiplexing capability, high specificity and sensitivity, and our expertise in the field. Additionally, the method requires minimal sample input, making it particularly suitable for precious samples such as breast milk, while its broad dynamic range allows for the detection of both low- and high-abundance analytes within the same assay.

### 2.4. Ethical Principles

The research adhered to the ethical guidelines outlined in the Declaration of Helsinki by the World Medical Association concerning medical investigations involving human participants. Approval for the study was granted by the Research Ethics Commission of the University of Medicine and Pharmacy “Grigore T. Popa” Iasi, Romania (IRB number: 211/2022).

Participation in the study was entirely voluntary and was conducted following the provision of informed consent. All participants provided their explicit agreement to partake in the study and consented to the publication of its findings in a specialized journal by signing the informed consent form.

### 2.5. Statistical Analysis

Statistical analysis was performed using IBM SPSS Statistics software, version 20. The distribution of variables was examined using the Kolmogorov–Smirnov test. Spearman’s correlation tests were applied to assess the relationships between variables, with the *p*-value representing the α-significance level. A significance level of less than 0.05 indicates a less than 5% probability that the observed result occurred by chance, thus reducing the likelihood of false positives. The strength of correlation was categorized based on the r value as follows: 0–0.29 (poor correlation), 0.3–0.49 (moderate correlation), and 0.5–1 (strong correlation). Group comparisons were performed using independent sample t-tests or paired-sample t-tests. Receiver operating characteristic (ROC) analysis, including the assessment of the area under the curve (AUC), was conducted to evaluate the sensitivity and specificity of the biomarkers in predicting infection status. The conclusions of this study were derived from the results of the statistical analyses, which were conducted on data from the entire study cohort as well as the three distinct subgroups.

## 3. Results

### 3.1. Study Group Characteristics

The assessment of the required number of study participants was conducted using a sample size calculator. The analysis indicated that at least 59 surveys were necessary to attain the established statistical criteria.

The statistical power of this research was calculated using Python software 3.4, incorporating key parameters such as sample size (N) = 60, effect size (f) = 0.5, and significance level (α) = 0.05. The analysis was conducted using Cohen’s f formula and power analysis methods, including *t*-tests. The resulting statistical power was approximately 0.968 (96.8%), indicating a high probability of correctly detecting a true effect if one exists. As a power level of 0.80 (80%) or higher is generally considered acceptable, the study is well-powered.

The study included a cohort of 60 lactating mothers, divided into three distinct groups as previously described: 24 participants who had recently contracted SARS-CoV-2, 26 participants who had received anti-SARS-CoV-2 vaccination without a prior history of COVID-19 up to the sampling period, and a control group consisting of 10 unvaccinated mothers with no history of COVID-19. To verify the integrity of the control group and confirm the absence of asymptomatic COVID-19 cases, screening for anti-SARS-CoV-2 IgG antibodies in breast milk was performed, which showed no presence of antibodies. In contrast, anti-SARS-CoV-2 IgG antibodies were detected in the breast milk samples from both sampling periods in the other two groups.

The demographic characteristics of all mothers in the study group, categorized based on their respective groups and inclusion criteria, are presented in the three tables included in the Supplementary Materials (Tables S1–S3).

Maternal ages ranged from 29 to 44 years, while the ages of breastfed infants ranged from 2 to 34 months. Table 3 presents the statistical parameters related to the ages of both mothers and infants for the entire study cohort and the subgroups. All participants resided in urban areas and were employed in a variety of occupational fields.

None of the children were born prematurely, experienced birth complications, or had any inflammatory or infectious diseases at the time of study enrollment or during sample collection. The distribution of characteristics among the groups of mothers in the study is presented in Table 4.

### 3.2. Anti-Inflammatory Proteins Assessment

All the tested markers, chitinase 3-like 1, furin, granzyme B, lactoferrin, lactadherin, and tenascin C were detected in the breast milk of every mother in the batch. The statistical parameters relevant to describing the variability of the obtained data are presented in Table 5.

The concentrations of F, GB, LF, and LD in breast milk were lower than the serum values provided by the manufacturers. However, the C3-1 concentration in breast milk exceeded the standard serum value in 42 (70%) of the participants across the entire study group, including 7 from the control group, 16 from the infected group, and 19 from the immunized group. Additionally, the concentration of TC exceeded the serum threshold in 9 (15%) of the mothers in the study cohort. For a clearer visualization of the variable distribution among the study groups, we conducted a comparison of the studied markers across all three study groups Table 6.

The distribution of variable scores across the sample was analyzed, and the test results indicated statistical significance (*p* < 0.05), suggesting that the variable’s distribution deviates significantly from normality. All variables followed an abnormal distribution, as confirmed by the Kolmogorov–Smirnov test (Table 3 and Table 5).

Spearman’s correlation analysis applied to the entire group revealed that C3-1 levels were correlated with F concentrations and the child’s age; C3-1 levels were correlated with maternal parity; F concentrations correlated with GB, TC, IL-6, the child’s age, and maternal parity; GB correlated with TC and IL-6; LF correlated with maternal immunization or infection status; LD concentrations correlated with TC, LF, the child’s age, and maternal infection or vaccination status; and TC correlated with IL-6 and the child’s age (Table 7). The correlations not mentioned in the text or Table 6 were not statistically significant.

The results of the One-Sample t-test indicated that the concentration of C3-1 in breast milk was significantly higher than the standard serum value provided by the kit manufacturer, while the concentrations of GB and F were significantly lower than the standard serum value (Table 8).

**Figure 2:**
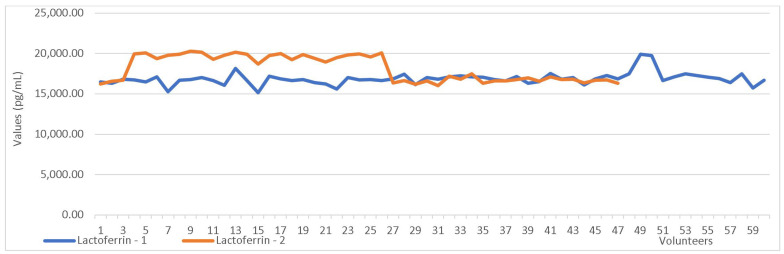
Lactoferrin values in the first vs. the second sampling.

**Figure 3:**
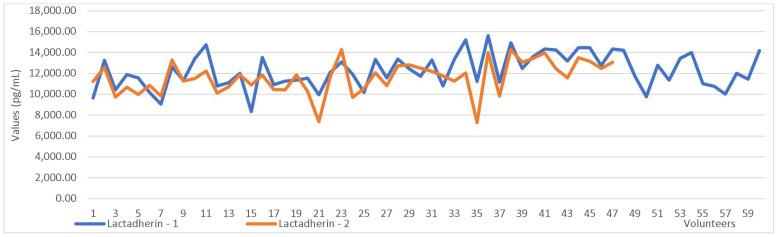
Lactadherin values in the first vs. the second sampling.

The paired-sample test, which compared the first and second collection values, revealed significant differences in the concentrations of LF and LD between the two sampling time points (Table 9, Figure 2 and Figure 3).

**Figure 4:**
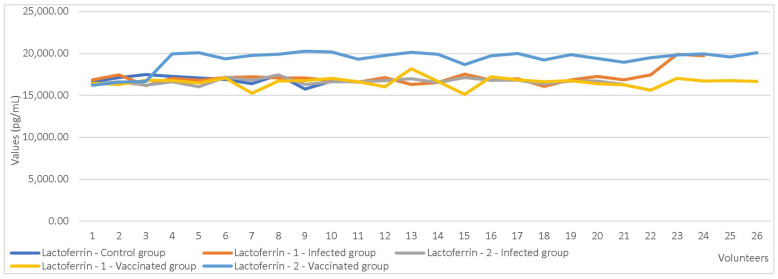
Comparisons of lactoferrin values between the 3 study groups.

**Figure 5:**
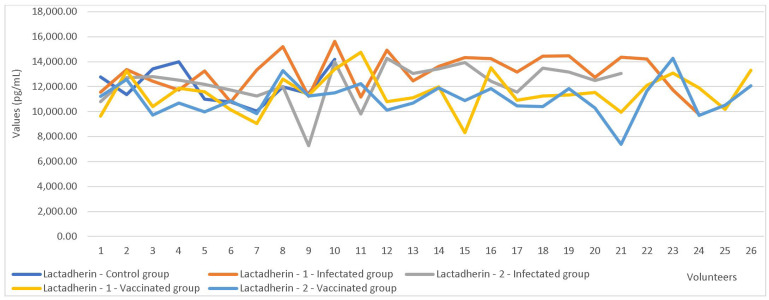
Comparisons of lactadherin values between the 3 study groups.

An independent sample t-test was used to compare markers’ values based on maternal parity showing C3-1 (*p* = 0.017) and F (*p* = 0.039) registered statistically significant differences. Furthermore, the test also yielded significant results when comparing C3-1 values based on the mode of delivery (*p* = 0.027). Statistically significant differences were also observed in LF concentrations based on maternal immunization status (*p* < 0.001). Comparison tests showed significant differences in LF (*p* = 0.001) and LD (*p* = 0.006) values between immunized mothers and those who were infected (Table 10, Figure 4 and Figure 5).

**Figure 6:**
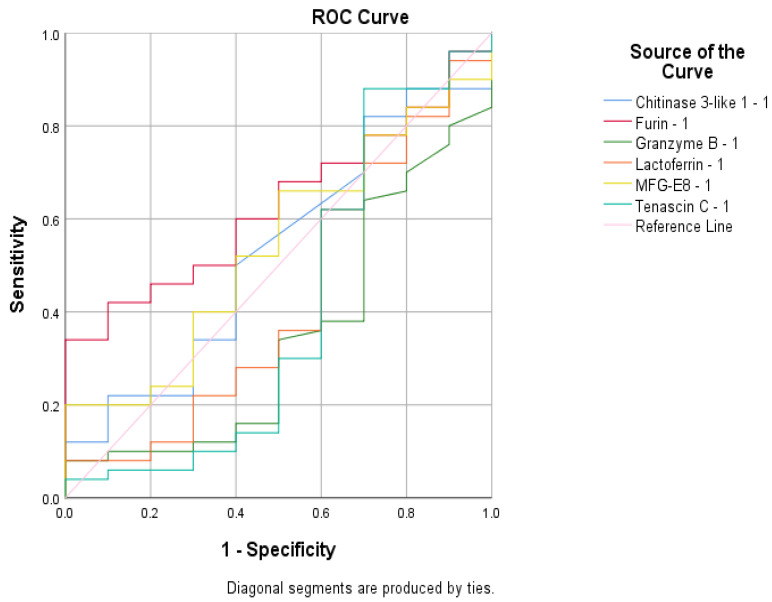
The ROC curve generated to predict the presence of antibodies.

**Figure 7:**
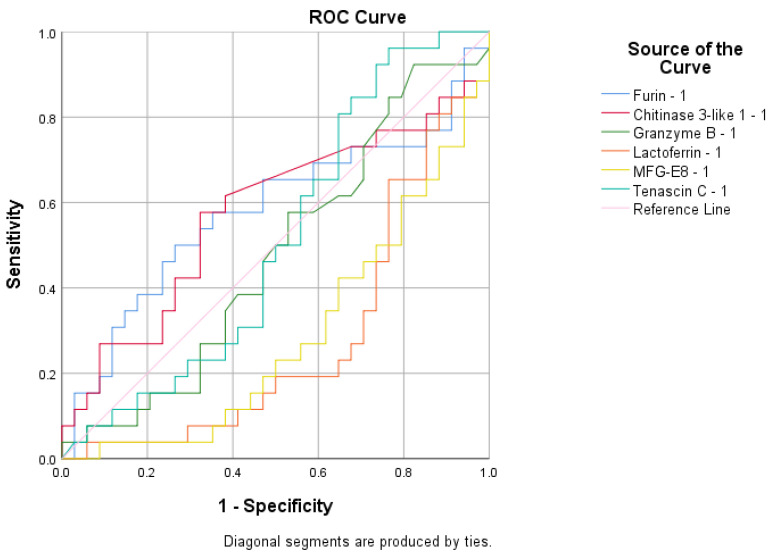
The ROC curve generated to predict immunization status.

**Figure 8:**
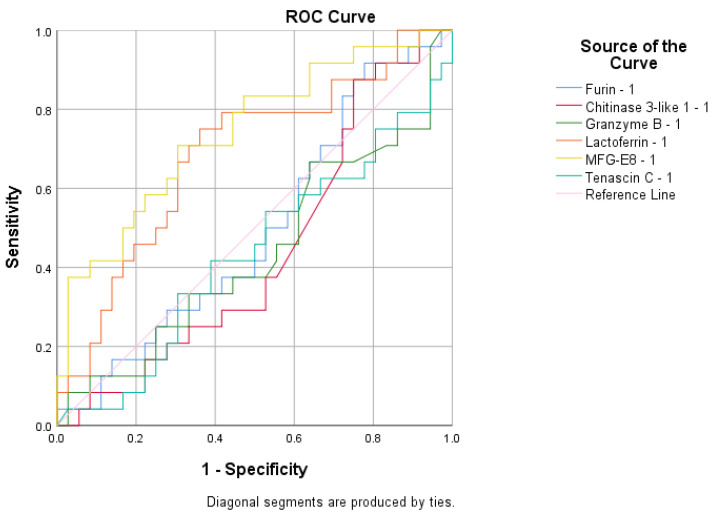
The ROC curve generated to predict the infection status.

To assess the sensitivity and specificity of the biomarkers in predicting infection status or vaccination response, several ROC analyses were performed. F was found to be a poor predictor of antibody presence with an area under the curve (AUC) value of 0.630 (Figure 6). None of the tested variables were able to predict immunization status, as their AUC values were below 0.6 (Figure 7). The biomarker that best predicted infection status was LD, with an AUC value of 0.742 (Figure 8).

## 4. Discussion

In our study, we compared the immune response triggered by natural SARS-CoV-2 infection and COVID-19 vaccination, providing valuable insights into the type and quality of immunity transferred to the infant through breast milk.

The investigation into the biomarkers present in the breast milk of mothers infected with SARS-CoV-2 or vaccinated against COVID-19 was driven by the need to understand how maternal immune responses are transferred to infants. This research holds significant implications for infant immunity, public health, and the long-term clinical outcomes for infants exposed to COVID-19-related immune factors through breastfeeding. By studying these immune components, we aimed to better comprehend how maternal infection or vaccination might influence neonatal health in the context of COVID-19.

The primary objective of this study was to compare the levels of the tested biomarkers between two distinct groups—those infected with SARS-CoV-2 and those vaccinated against COVID-19—in order to assess differences in antimicrobial protein profiles. This comparison sought to determine whether infection or vaccination induced more robust or effective antimicrobial responses in breast milk, explored the potential protective effects these bioactive proteins might have on breastfeeding infants, particularly regarding the prevention or mitigation of COVID-19 infection, and evaluated how maternal infection or vaccination influenced the transfer of passive immunity to infants via breast milk.

Furthermore, the study intended to assess whether breastfeeding provides additional immune benefits during the COVID-19 pandemic, as indicated by changes in biomarker levels, and aimed to provide evidence to guide public health recommendations for breastfeeding practices among mothers who have been infected with SARS-CoV-2 or vaccinated against COVID-19.

Newborns possess an immature immune system, making them highly reliant on maternal antibodies, antimicrobial compounds, and innate immunity for protection against infections. Consequently, breast milk is rich in immunomodulatory substances, antibodies, and antimicrobial molecules that provide essential protection [46,47,48,49,50].

With the onset of the COVID-19 pandemic, it became essential to explore how the immune profile of breast milk changes in mothers who have either been infected with SARS-CoV-2 or vaccinated. Our identification of biomarkers such as C3-1, F, GB, LF, LD, and TC in this context revealed that maternal protection is effectively transferred to the child, significantly influencing neonatal antimicrobial defense.

LF and LD are prominent bioactive proteins in breast milk recognized for their antimicrobial and anti-inflammatory properties. LF has gained particular attention for its potential effectiveness against SARS-CoV-2 due to its dual mechanism of action [51,52].

Our investigation of LF levels in the breast milk of mothers exposed to SARS-CoV-2 or vaccinated revealed its critical role in enhancing passive immunity for infants, thereby offering protection against infections, including COVID-19. This was evidenced by a direct correlation and a significant difference in LF levels relative to the mother’s infection or vaccination status. LF levels were found to be elevated in the breast milk of both vaccinated and, to a greater extent, infected mothers compared to the control group.

In our study, higher levels of LF were also associated with the presence of IgG antibodies against SARS-CoV-2 in the serum of participants. The increased LF levels in immunized mothers reflect heightened immune activation, antimicrobial defense mechanisms, and an adaptive response aimed at enhancing protection for both the mother and the nursing infant through breast milk. This rise in LF levels observed by us can be attributed to various immunological and physiological factors. Following vaccination, especially against viral pathogens like SARS-CoV-2, there is an elevated production of immune components, including LF. As an acute-phase protein with antimicrobial properties, LF levels surge in response to immune activation, providing enhanced protection to both the mother and the breastfeeding infant. Vaccination has been shown to modify the composition of breast milk by increasing immune factors that support the infant’s immunity [53]. Additionally, LF plays a crucial role in anti-inflammatory and immunomodulatory activities, contributing to immune regulation and maintaining homeostasis [16]. This increase in LF provides passive immunity to the infant, offering an additional layer of protection. Elevated LF levels likely reflect the maternal immune response aimed at transferring enhanced protection to the infant, especially against viral infections such as COVID-19 [54,55].

The findings of the present study suggested that LD levels in breast milk may help shield infants from infections, including COVID-19. These findings were supported by direct correlations and significant differences in LD concentrations based on the mother’s infection or vaccination status, with higher levels observed in the breast milk of both vaccinated and infected mothers compared to the control group.

Additionally, the ROC curve analysis revealed that, in this study, LD was the most effective predictor of infection status in our cohort. Elevated LD levels, surpassing the cutoff value derived from ROC analysis sensitivity and specificity, were indicative of SARS-CoV-2 infection in the study group.

Our analysis also showed a correlation between LD levels and the severity of maternal symptoms. Higher concentrations of LD were associated with more severe symptoms, suggesting that elevated LD levels reflect the body’s response to a more significant immune challenge. This increase in LD may serve to modulate inflammation and provide additional protection against pathogens [56]. LD levels are known to rise during inflammatory conditions, where it facilitates the clearance of apoptotic cells, thereby reducing excessive inflammation [16].

Severe symptoms are typically linked to a higher viral load or more intense infection, prompting the body to upregulate LD production as a protective measure. This increase in LD production may enhance the antimicrobial defense provided to the infant, especially when the mother is facing a severe infection. Studies have documented the antimicrobial role of LD, demonstrating its ability to block viral entry by binding to viral particles [53].

In previous research, we have observed that severe symptoms often coincide with increased levels of pro-inflammatory cytokines, such as IL-6 [57]. Furthermore, recent studies have shown that LD expression is upregulated in response to these inflammatory cytokines, which aligns with its function in resolving inflammation and facilitating tissue repair [55]. During severe infections, the maternal immune system adapts to protect both the mother and the infant, potentially increasing the concentration of immune factors like LD in breast milk to enhance the infant’s passive immunity [58].

An important observation in our study was the significant reduction in LF and LD concentrations during the second sampling in both vaccinated and infected mothers. This decrease suggests a trend of normalization following the resolution of viral or post-vaccination effects, further supporting the dynamic nature of immune responses in breast milk.

TC is an extracellular matrix protein that plays a crucial role in tissue repair and immune regulation [58,59,60]. In the present study, TC levels were found to be directly correlated with inflammation, as indicated by IL-6 levels. As inflammation increased, so did TC concentrations, suggesting its role in protecting infants from COVID-19 through breast milk.

Our statistical analysis demonstrated that GB levels were directly correlated with the anti-inflammatory response; as IL-6 levels increased, GB concentrations also rose. This finding supports the known anti-inflammatory effects of GB [61,62,63,64], which were evident in our study cohort. Although GB was present in the breast milk at significantly lower concentrations than standard serum levels, its correlation with IL-6 suggests that it may contribute to anti-inflammatory and antimicrobial protection in breastfeeding infants [61,62,63,64].

Understanding the roles of chitin and chitinase-like proteins is essential for advancing disease prevention and treatment strategies, particularly in the context of COVID-19. In our study, C3-1 levels in breast milk were significantly higher than the standard serum values provided by the kit manufacturer, with most volunteers exhibiting elevated levels. However, despite these elevated concentrations, no significant differences in C3-1 levels were observed in relation to SARS-CoV-2 infection or vaccination status. The absence of significant variation within the study group limits our ability to definitively assess the role of C3-1 in maternal immune activation and its potential impact on inflammatory processes [65,66,67,68] in breastfeeding infants during infection or vaccination.

F is an enzyme that plays a critical role in activating certain viral proteins [69,70], including the spike protein of SARS-CoV-2 [71,72]. Additionally, host cell F is involved in the activation of various bacterial toxins, such as those produced by *Bacillus anthracis* [73,74], *Corynebacterium diphtheriae* [74], *Pseudomonas aeruginosa* [75], Shiga toxins [75], and the dermonecrotic toxins of *Bordetella* species [76,77].

Although F levels in our study group were significantly lower than the standard serum values, F activity was found to be associated with the inflammatory response, as F concentrations increased in tandem with IL-6 levels. Furthermore, the ROC analysis suggested that F levels could serve as a predictive marker for immunization or infection status. This reinforces the understanding of F’s antimicrobial role during lactation, providing valuable insight into the viral defense mechanisms in breastfed infants.

F was a useful predictor of the presence of anti-SARS-CoV-2 IgG antibodies in breast milk in our study, owing to its involvement in viral protein processing, immune activation, enhanced antigen presentation, and its correlation with inflammatory responses. This underscored the interrelated roles of proteases and antibodies in the maternal immune response. During infection or after vaccination, F activity is upregulated as it plays a crucial role in cleaving the viral spike protein, facilitating viral entry, and triggering an immune response. The increased presence of F in our research might indicate an active immune process, which corresponded with the production of specific antibodies, such as IgG, against SARS-CoV-2.

Elevated F activity may enhance antigen processing, thereby stimulating a more robust humoral response and promoting the generation of IgG antibodies. This connection may explain why higher levels of F are predictive of the presence of anti-SARS-CoV-2 IgG in breast milk [78,79]. In the context of SARS-CoV-2 infection or vaccination, the maternal immune system intensifies its defense mechanisms, including the production of proteases like F. Elevated levels of F may thus serve as an indicator of an enhanced immune state, aligning with the production of specific antibodies against the virus. This association likely reflects a coordinated immune response, wherein F expression is part of the broader activation of the immune system that leads to the generation of IgG antibodies [80].

Additionally, in our study, F levels were correlated with pro-inflammatory markers in our study, which were elevated during infection or post-vaccination. Given that inflammation can promote immune activation and antibody production, higher F concentrations may reflect an underlying immune process that also contributes to increased IgG levels in breast milk [55].

In our research, the concentrations of C3-1 and F in breast milk were found to vary with maternal parity, with levels decreasing as the number of births increased. Specifically, the excretion of F and C3-1 in breast milk was higher in mothers with firstborns. The observed decrease in C3-1 and F levels with increasing maternal parity may be linked to immune and physiological adaptations that occur with subsequent pregnancies and births. This finding is consistent with previous research indicating that first pregnancies often involve a stronger immune response as the maternal immune system encounters fetal antigens for the first time. As a result of our analysis, primiparous mothers tended to experience heightened inflammatory and immune responses compared to multigravida women, which may explain the observed variations in immune-modulating proteins in breast milk across different pregnancies [81,82].

Following the first pregnancy and childbirth, the mother’s mammary glands undergo structural transformations, including tissue remodeling, which has been associated with the production of proteins such as C3-1. These proteins contribute to tissue repair and immune modulation. In subsequent pregnancies, the need for such intense tissue adaptation may diminish, leading to lower levels of remodeling-related proteins [83,84,85].

Research has indicated that firstborns may receive breast milk with higher concentrations of immune-modulating proteins, potentially reflecting the mother’s initial investment in supporting neonatal immunity [16,86,87].

In our study, variations in C3-1 levels were observed based on the mode of delivery, with mothers who underwent vaginal delivery showing lower concentrations of C3-1 in their breast milk compared to those who had a cesarean section. This difference may be attributed to the distinct inflammatory responses associated with natural versus cesarean deliveries. Cesarean sections generally involve greater surgical stress and immune activation, which can trigger a more pronounced inflammatory response when compared to vaginal deliveries. This increased inflammation could elevate the levels of certain immune-related proteins, including C3-1, known for its involvement in inflammation and immune modulation [67].

Furthermore, C3-1 is frequently associated with wound healing and tissue repair, processes that are more prominent following cesarean delivery. The body may produce higher levels of C3-1 to support recovery and manage the heightened inflammatory response induced by the surgery. This could help explain why mothers who undergo cesarean sections might have increased concentrations of C3-1 in their breast milk [67].

The correlation between C3-1, F, LD, and TC levels in the breast milk of our mothers and the age of the infant can be attributed to several factors related to the infant’s evolving nutritional and immunological needs. These findings align with the broader understanding that breast milk undergoes dynamic changes in its bioactive composition, adapting to the infant’s developmental stages and providing tailored support to meet the infant’s shifting physiological demands.

The composition of breast milk undergoes dynamic changes to meet the evolving developmental needs of the infant. In our study, the observed increase in concentrations of C3-1, F, LD, and TC in breast milk as the infant aged can be attributed to several factors related to the infant’s maturing immune system and ongoing maternal physiological adaptations. These findings underscore the dynamic nature of breast milk and its role in adapting to the infant’s changing developmental and immunological requirements. The increase in these specific proteins reflects a maternal strategy designed to continue providing enhanced protection and support as the infant progresses through various stages of early life.

As infants grow, their immune system gradually matures, although it remains in a transitional phase for an extended period. During this phase, the demand for immune-modulatory and protective factors in breast milk may intensify. Proteins such as C3-1, F, LD, and TC may be upregulated to offer continued protection as the infant encounters more environmental pathogens and dietary antigens, particularly with the introduction of solid foods. This adjustment aligns with the adaptive function of breast milk in responding to the infant’s exposure to new antigens and microorganisms [16].

Breast milk composition is not static; it adjusts in response to both maternal and infant health changes. The prolonged lactation phase may induce sustained maternal immune adaptations, leading to the increased secretion of immune-related proteins, including F and TC, which contribute to antimicrobial defenses. This ongoing maternal response becomes more pronounced as the infant ages, representing an adaptive mechanism to enhance protection against greater pathogen exposure during later infancy [84,85].

As infants grow older and gain more mobility, their exposure to various pathogens increases, highlighting the importance of enhanced immune protection. Elevated levels of bioactive proteins in breast milk may serve as a protective response by the mother, bolstering the infant’s immune system. For example, increased levels of LD have been associated with enhanced antimicrobial properties, while TC and C3-1 are linked to anti-inflammatory responses, which become increasingly critical as the infant’s exposure to infectious agents rises [87,88].

As the infant’s nutritional and developmental requirements change, the concentrations of specific bioactive components in breast milk may adjust accordingly. Proteins such as F and C3-1, involved in tissue repair and growth, are particularly important during periods of rapid development. Therefore, the increased levels of these proteins may reflect a maternal adaptation to support the infant’s ongoing growth needs [85,88].

Other comparisons made by us revealed that maternal vaccination may influence the immune composition of breast milk differently from natural infection. Understanding these differences is critical for evaluating how breastfeeding mothers provide passive immunity and guiding recommendations on maternal vaccination during breastfeeding to optimize infant health.

Breastfeeding remains a vital means of transferring immunity from mother to child, particularly for infants with developing immune systems. Our study identifies specific proteins such as TC, GB, and C3-1, which provide insight into how maternal COVID-19 infection or vaccination affects immune protection conferred to the infant. The long-term health outcomes of infants born during the COVID-19 pandemic are still being studied, but determining the presence and role of these immune factors in breast milk offers valuable information on how early exposure to maternal antibodies and immune proteins shapes infant immunity and development.

Our study identified differences in lactoferrin and lactadherin levels between vaccinated mothers and those who had been infected. Additionally, variations in furin levels were observed between both infected and vaccinated mothers compared to the control group. Furthermore, chitinase 3-like 1 levels were elevated in mothers who delivered via cesarean section, while primiparous mothers exhibited higher levels of both furin and chitinase 3-like 1 (Table 11). All other unmentioned variables did not show statistically significant differences between the analyzed groups.

Our findings have significant implications for public health recommendations regarding breastfeeding practices during the pandemic and post-vaccination. The elevated immune components observed after vaccination support the promotion of vaccination among breastfeeding mothers, as it may enhance infant protection. Furthermore, these findings provide reassurance about the safety and benefits of continuing breastfeeding during maternal infection with SARS-CoV-2.

## 5. Conclusions

The analysis of a set of bioactive compounds within the breast milk of infected or vaccinated mothers showed an enhancement of passive immunity, thus strengthening the infants’ immune system and potentially reducing the risk of infections, including of COVID-19.

We observed that chitinase 3 like-1’s milk levels increased with the infant’s age, but decreased with maternal parity and varied by delivery type, significantly exceeding normal serum concentrations.

Lactoferrin level was highest in infected mothers, followed by vaccinated and control groups, and it was higher in immunized volunteers. Lactadherin also showed higher levels in infected mothers, followed by vaccinated mothers, and increased with the infant’s age. Furin levels increased with IL-6 and the infant’s age, while decreasing with maternal parity. Granzyme B levels rose with IL-6. Tenascin C levels rose with IL-6 and decreased with the child’s age. Lactoferrin and lactadherin decreased from the first to the second milk collection. Furin predicted antibody presence, while lactadherin indicated infection status.

These findings underscore the complex interaction between maternal immune status, lactational adjustments, and infant needs, highlighting breastfeeding’s significant antimicrobial benefits, including protection against SARS-CoV-2. This research provides crucial data to guide evidence-based recommendations on breastfeeding safety during and after maternal infection or vaccination.

## Figures and Tables

**Table 1:** Human milk bioactive components.

Components		Function	References
Immunoglobulins	IgA/sIgA	Antimicrobial	[3,4,5,6,7,8,9,10]
	IgG	Antimicrobial	[4,6,8,9,10,11,12]
	IgM	Antimicrobial	[3,6,9]
Oligosaccharides and glycan	HMOs	Prebiotics	[9,11,13,14,15]
Mucins	MUC1	Antimicrobial	[16]
	MUC4	Antimicrobial	[16]
Cytokine inhibitors	TNF-R I and II	Anti-inflammatory	[16]
Antimicrobial factors	Lactoferrin	Antimicrobial, anti-oxidant, anti-inflammatory	[16,17]
	Lactadherin	Antimicrobial, anti-oxidant, anti-inflammatory	[16,18]
Metabolic factors	Adiponectin	Anti-inflammatory	[19]
	Leptin	Regulation of energy conversion and infant weight; Regulation of appetite	[19]
	Ghrelin	Regulation of energy conversion; Control of infant weight; Role in maintaining harmonious development.	[19]

Abbreviation: Ig = immunoglobulin, HMO = human milk oligosaccharides.

**Table 2:** Study group inclusion and exclusion criteria.

Inclusion criteria	Breastfeeding mothers
	Immunization with two doses of mRNA vaccine after birth **OR**
	Onset of SARS-CoV-2 infection postbirth in the last month prior to study inclusion **OR**
	Positive result for RT-PCR/antigen postbirth in the last month prior to study inclusion **OR**
	Absence of anti-SARS-CoV-2 IgG antibodies in breast milk
	Apparent good health at the time of milk sample collection
	At any time from the start of breastfeeding
	Direct nursing or expressed breast milk
	Providing and signing informed consent
Exclusion criteria	Participants without verified vaccination records
	Participants without records of RT-PCR/antigen results
	Unrelated acute illnesses (e.g., mastitis)
	Clear signs of infection or inflammatory diseases
	Insufficient or contaminated milk samples
	Samples collected outside the designated time frame
	Improper methods of sampling
	Vaccination or infection at inappropriate time
	Incomplete vaccination scheme
	COVID-19 vaccination or infection during pregnacy

**Table 3:** Statistical parameters regarding the ages of the study group.

Study Group		Entire Group		Infected Mothers		Immunized Mothers		Control Group	
Category		Mother’s Age (Years)	Child’s Age (Months)	Mother’s Age (Years)	Child’s Age (Months)	Mother’s Age (Years)	Child’s Age (Months)	Mother’s Age (Years)	Child’s Age (Months)
Median		32.50	11.50	32.00	12.00	33.00	12.00	32.50	9.00
Std. Deviation		2.58	9.06	2.90	8.68	2.44	10.21	2.13	6.62
Variance		6.66	82.12	8.41	75.28	5.98	104.27	4.54	43.78
Range		10	33	10	31	9	33	7	22
Percentiles	25	31.00	5.25	31.00	6.25	31.00	4.00	30.00	4.50
	50	32.50	11.50	32.00	12.00	33.00	12.00	32.50	9.00
	75	35.00	19.75	36.00	20.75	35.00	20.50	33.25	13.75
IQR		4.00	14.50	5.00	14.25	4.00	16.50	3.25	9.25
*p*		0.002	0.039	0.002	0.004	0.001	0.000	0.001	0.000

*p =* Kolmogorov–Smirnov statistic coefficient of distribution of variables in the entire study group.

**Table 4:** Distribution of characteristics among the groups of mothers in the study.

Study Group		Entire Group Nb (Percent)	Vaccinated Mothers Nb (Percent)	Infected Mothers Nb (Percent)	Control Group Nb (Percent)
Births number	Primiparous	27 (45%)	17 (65.4%)	8 (33.3%)	2 (20%)
	Multiparous	33 (55%)	9 (34.6%)	16 (66.7%)	8 (80%)
Birth type	Natural birth	21 (35%)	7 (26.9%)	8 (33.3%)	4 (40%)
	Cesarean section	39 (65%)	19 (73.1%)	16 (66.7%)	6 (60%)
Vaccine type	Pfizer-BioNTech vaccine		23 (88.5%)	16 (66.7%)	
	Moderna vaccine		3 (11.5%)		
	No vaccine			8 (33.3%)	10 (100%)
Post-vaccination adverse reactions	Mild		17 (65.4%)		
	Moderate		4 (15.4%)		
	None		5 (19.2%)		
Severity form	Mild			13 (54.2%)	
	Moderate			9 (37.5%)	
	Asymptomatic			2 (8.3%)	

**Table 5:** Statistical parameters regarding the tested biomarkers.

Statistical Parameters		C3-1 1 (pg/mL)	C3-1 2 (pg/mL)	F 1 (pg/mL)	F 2 (pg/mL)	GB 1 (pg/mL)	GB 2 (pg/mL)	LF 1 (pg/mL)	LF 2 (pg/mL)	LD 1 (pg/mL)	LD 2 (pg/mL)	TC 1 (pg/mL)	TC 2 (pg/mL)
Median		115,790	116,316	5393	5169	26.82	21.99	16,815	17,464	11,993	11,743	3867	3584
Range		3,319,616	687,667	79,688	76,589	200	309	4771	4244	7292	6995	123,726	123,818
P	25	96,805	84,984	3674	3547	18.20	16.95	16,535	16,623	11,116	10,527	2139	2342
	50	115,790	116,316	5393	5169	26.82	21.99	16,815	17,464	11,993	11,743	3867	3584
	75	243,135	231,832	8852	9936	48.84	37.80	17,104	19,805	13,415	12,710	10,223	8050
IQR		146,329	146,847	5177	6388	30	20	568	3182	2299	2184	8084	5708
*p*		0.000	0.000	0.000	0.000	0.000	0.000	0.000	0.000	0.02	0.02	0.000	0.000

*p =* Kolmogorov–Smirnov statistic coefficient of distribution of variables in the study group. Abbreviations: P = percentiles; C3-1 = chitinase 3-like 1; F = furin; GB = granzyme B; LF = lactoferrin; LD = lactadherin; TC = tenascin C; 1 = first sampling; 2 = second sampling.

**Table 6:** Comparison of statistical parameters of studied biomarkers between the study groups.

Study Groups	Statistical Parameters		C3-1 1	C3-1 2	F 1	F 2	GB 1	GB 2	LF 1	LF 2	LD 1	LD 2	TC 1	TC 2
G1	Median		146,495	128,216	6618	5055	26	23	16,679	19,779	11,442	10,858	3688	3810
	P	25	91,505	69,433	3393	3410	18	17	16,388	19,286	10,345	10,253	2404	2624
		50	146,495	128,216	6618	5055	26	23	16,679	19,779	11,442	10,858	3688	3810
		75	299,741	253,665	10,559	10,099	42	40	16,823	19,954	12,729	11,866	7778	6871
G2	Median		115,790	115,790	5142	5687	23	22	17,014	16,651	13,299	12,515	3749	3397
	P	25	97,936	115,790	3812	3712	16	18	16,774	16,346	11,727	11,651	1751	1900
		50	115,790	115,790	5142	5687	23	22	17,014	16,651	13,299	12,515	3749	3397
		75	217,500	214,867	8842	14,411	47	45	17,247	16,799	14,344	13,296	7548	10,020
G3	Median		115,790		4581		43		16,977		11,717		8766	
	P	25	74,457		3414		20		16,584		10,950		1248	
		50	115,790		4581		43		16,977		11,717		8766	
		75	259,512		6396		88		17,321		13,562		21,537	

Abbreviations: G1 = Vaccinated mothers; G2 = infected mothers; G3 = control group; P = percentiles; C3-1 = chitinase 3-like 1; F = furin; GB = granzyme B; LF = lactoferrin; LD = lactadherin; TC = tenascin C; 1 = first sampling; and 2 = second sampling.

**Table 7:** Spearman’s correlation.

Parameters		C3-1 1	C3-1 2	F 1	F 2	GB 1	GB 2	LF 1	LF 2	LD 1	LD 2	TC 1	TC 2
IL-6	ρ	0.206	0.175	0.319	0.314	0.442	0.476	0.087	−0.182	−0.031	0.021	0.361	0.320
	*p*	0.120	0.238	0.015	0.032	0.001	0.001	0.515	0.222	0.817	0.890	0.005	0.028
Antibodies	ρ	−0.036		−0.168		0.195		0.098		−0.052		0.124	
	*p*	0.783		0.200		0.135		0.456		0.695		0.345	
Affiliation with the study subgroup	ρ	−0.116	0.076	−0.171	0.098	0.109	0.021	0.337	0.726	0.264	0.475	0.036	−0.098
	*p*	0.378	0.612	0.193	0.513	0.407	0.891	0.008	0.000	0.041	0.001	0.783	0.513
Child’s age	ρ	0.227	0.318	0.387	0.447	−0.208	−0.090	0.183	0.161	0.263	0.166	−0.439	−0.344
	*p*	0.081	0.029	0.002	0.002	0.110	0.545	0.161	0.278	0.042	0.265	0.000	0.018
Parity	ρ	−0.190	−0.399	−0.320	−0.224	−0.053	0.067	0.063	−0.171	−0.067	0.009	0.052	0.064
	*p*	0.147	0.005	0.013	0.129	0.686	0.652	0.633	0.250	0.612	0.950	0.692	0.668
Mother’s age	ρ	−0.053	−0.218	−0.092	−0.095	−0.065	0.038	0.052	0.067	−0.097	−0.127	−0.003	0.093
	*p*	0.686	0.141	0.486	0.526	0.621	0.799	0.696	0.655	0.461	0.394	0.983	0.534
Birth	ρ	0.040	0.138	0.013	−0.015	0.007	0.145	0.017	−0.062	−0.017	0.077	0.021	0.151
	*p*	0.764	0.354	0.921	0.920	0.957	0.332	0.897	0.678	0.897	0.605	0.872	0.310
Vaccine	ρ	0.006	0.019	−0.151	0.047	0.116	0.115	0.233	−0.381	0.015	0.118	0.042	−0.047
	*p*	0.962	0.900	0.249	0.754	0.379	0.441	0.073	0.008	0.911	0.431	0.750	0.754
Symptoms	ρ	−0.061	0.030	−0.090	−0.046	−0.043	−0.004	−0.010	−0.257	0.137	0.288	0.047	−0.127
	*p*	0.675	0.842	0.536	0.759	0.764	0.976	0.943	0.081	0.344	0.049	0.748	0.395

Legend: yellow marked values are statistically significant. 1 = first sampling. 2 = second sampling. C3-1 = chitinase 3-like 1; F = furin; GB = granzyme B; LF = lactoferrin; LD = lactadherin; and TC = tenascin C.

**Table 8:** Comparisons of the values obtained with standard serum values.

	*p*	Mean Difference
Chitinase 3-like 1-1	0.025	134,661.53
Chitinase 3-like 1-2	0.003	76,933.47
Furin-1	0.000	−18,226.66
Furin-2	0.000	−15,196.28
Granzyme B-1	0.000	−4328.40
Granzyme B-2	0.000	−4326.83

1 = first sampling. 2 = second sampling.

**Table 9:** Comparisons of the values obtained in the two samplings.

	Standard Deviation	*p*
Lactoferrin-1 vs. Lactoferrin-2	1742.30	0.000
Lactadherin 1 vs. Lactadherin-2	1262.30	0.000

1 = first sampling. 2 = second sampling.

**Table 10:** Statistically significant differences throughout the study group.

Grouping Variable	Dependent Variables	*p*
Mother’s parity	Furin	0.021
	Chitinase 3-like 1	0.017
Delivery mode	Chitinase 3-like 1	0.027
Immunization status	Lactoferrin	0.000
Vaccinated mother group vs. infected mother group	Lactoferrin	0.000
	Lactadherin	0.001

**Table 11:** Differences throughout the study groups.

Groups	Action	Parameters	Statistical Test	*p*
Vaccinated mothers vs. infected mothers vs. control group	Values decrease from vaccinated to control group	Lactoferrin	Spearman’s correlation	<0.05
Vaccinated mothers vs. infected mothers vs. control group	Values decrease from vaccinated to control group	Lactadherin	Spearman’s correlation	<0.05
Primiparous vs. multiparous mothers	Depending on mother’s parity the parameters decrease	Furin Chitinase 3-like 1	Independent sample *t*-test	<0.05
Natural births vs. C-section	Depending on delivery mode the parameters increase	Chitinase 3-like 1	Independent sample *t*-test	<0.05
Vaccinated mothers vs. non-vaccinated mothers	Depending on immunization status the parameters increase	Lactoferrin	Independent sample *t*-test	<0.05
Vaccinated mother group vs. infected mothers groups	Depending on group assignment the parameters increase	Lactoferrin Lactadherin	Independent sample *t*-test	<0.05
Vaccinated and infected mothers vs. control group	Values decrease in control group	Furin	ROC analyses	-
Infected mothers group vs. vaccinated mothers and control group	Values decrease in vaccinated mothers and control group	Lactadherin	ROC analyses	=

## Data Availability

The original contributions presented in this study are included in the article/Supplementary Material. Further inquiries can be directed to the corresponding author.

## Supplementary Materials

The following supporting information can be downloaded at https://www.mdpi.com/article/10.3390/cimb47030182/s1.
