# Supplementary material for: The Legacy of COVID-19 in Breast Milk: The Association of Elevated Anti-Inflammatory and Antimicrobial Proteins with Vaccination or Infection
Source: Curr Issues Mol Biol. 2025 Mar 11;47(3):182. doi: 10.3390/cimb47030182 (PMC11941678; doi:10.3390/cimb47030182)
Supplement: Supplementary file 1 [file cimb-47-00182-s001.zip › cimb-3479378-supplementary.pdf]

Supplementary Materials

Table S1. Characteristics of anti-SARS – CoV – 2 vaccinated mothers' group

ID No	Anti-RBD IgG	Child`s age (months)	Mother`s age (years)	Mother`s parity	Birth type	Vaccine type	Side effects after vaccination
1	+	9	30	1	Cesarean section	Pfizer	Local pain
2	+	23	31	1	Cesarean section	Pfizer	Local pain
3	+	4	38	1	Cesarean section	Moderna	Local pain
4	+	2	33	2	Cesarean section	Pfizer	Local pain
5	+	25	31	1	Cesarean section	Pfizer	Local pain
6	+	3	36	2	Cesarean section	Pfizer	Local pain
7	+	2	33	2	Natural birth	Pfizer	Absent
8	+	33	31	1	Cesarean section	Pfizer	Generalized muscle pain, fever, headache
9	+	12	36	2	Cesarean section	Pfizer	Local pain
10	+	35	31	1	Natural birth	Pfizer	Local pain
11	+	19	35	1	Cesarean section	Pfizer	Absent
12	+	4	34	2	Natural birth	Pfizer	Local pain
13	+	10	32	1	Natural birth	Pfizer	Generalized muscle pain, fever, headache
14	+	10	32	1	Cesarean section	Pfizer	Absent
15	+	18	29	1	Natural birth	Pfizer	Absent
16	+	20	35	2	Cesarean section	Pfizer	Local pain
17	+	12	35	2	Cesarean section	Pfizer	Generalized muscle pain, fever, headache
18	+	2	37	2	Cesarean section	Moderna	Local pain
19	+	11	34	1	Cesarean section	Pfizer	Local pain
20	+	4	32	1	Cesarean section	Pfizer	Local pain
21	+	3	30	1	Cesarean section	Pfizer	Local pain
22	+	22	30	1	Natural birth	Pfizer	Local pain
23	+	15	35	1	Natural birth	Pfizer	Absent
24	+	12	36	2	Cesarean section	Moderna	Local pain
25	+	17	34	1	Cesarean section	Pfizer	Local pain
26	+	34	32	1	Cesarean section	Pfizer	Generalized muscle pain, fever, headache

Table S2. Characteristics of SARS – CoV – 2 infected mothers' group

ID No	Anti-RBD IgG	Child`s age (months)	Mother`s age (years)	Mother`s parity	Birth type	Vaccine prior infection	Symptoms	Hospitalization	Oxygen therapy
1	+	4	37	2	Cesarean section	Yes	sore throat, asthenia	No	No
2	+	6	35	1	Cesarean section	Yes	significant asthenia, altered general condition, cough, sore throat	No	No
3	+	2	31	2	Natural birth	No	sore throat, fever, muscle pain	No	No

4	+	7	30	2	Cesarean section	Yes	sore throat, lack of smell/taste	No	No
5	+	3	32	2	Cesarean section	No	fever, muscle pain	No	No
6	+	11	30	1	Natural birth	Yes	significant asthenia, altered general condition, fever, cough, sore throat	No	No
7	+	6	36	3	Cesarean section	Yes	significant asthenia, altered general condition, fever	No	No
8	+	28	31	1	Cesarean section	Yes	lack of smell/taste	No	No
9	+	21	33	2	Natural birth	No	sore throat, muscle pain	No	No
10	+	16	35	2	Natural birth	Yes	lack of smell/taste	No	No
11	+	33	31	1	Natural birth	Yes	lack of smell/taste, fever, muscle pain	No	No
12	+	24	32	1	Cesarean section	No	significant asthenia, altered general condition, cough, sore throat, muscle pain	No	No
13	+	14	38	3	Natural birth	Yes	significant asthenia, altered general condition, cough, fever, muscle pain	No	No
14	+	9	32	1	Cesarean section	Yes	significant asthenia, altered general condition, cough, fever	No	No
15	+	20	36	2	Cesarean section	Yes	sore throat, muscle pain	No	No
16	+	13	29	2	Cesarean section	No	sore throat, fever	No	No
17	+	21	28	2	Natural birth	No	significant asthenia, altered general condition, cough, fever	No	No
18	+	13	32	2	Cesarean section	Yes	significant asthenia, altered general condition, cough, fever	No	No
19	+	16	38	2	Natural birth	Yes	sore throat, fever, muscle pain	No	No
20	+	4	36	2	Cesarean section	Yes	sore throat, lack of smell/taste	No	No
21	+	7	32	1	Cesarean section	Yes	significant asthenia, altered general condition, fever, muscle pain	No	No
22	+	8	31	1	Cesarean section	Yes	sore throat, fever, lack of smell/taste	No	No

23	+	8	36	2	Cesarean section	No	No simptoms	No	No
	+	26	35	1	Cesarean section	No	No simptoms	No	No

Table S3. Characteristics of the control group

**ID No**	**Anti-RBD IgG**	**Child`s age (months)**	**Mother`s age (years)**	**Mother`s parity**	**Birth type**
1	-	9	32	2	Natural birth
2	-	13	29	2	Cesarean section
3	-	24	33	2	Natural birth
4	-	9	34	2	Cesarean section
5	-	12	30	2	Cesarean section
6	-	5	31	1	Natural birth
7	-	2	33	3	Cesarean section
8	-	16	33	1	Natural birth
9	-	3	36	2	Natural birth
10	-	7	30	2	Natural birth
